# Canine Disorder Mirrors Human Disease: Exonic Deletion in *HES7* Causes Autosomal Recessive Spondylocostal Dysostosis in Miniature Schnauzer Dogs

**DOI:** 10.1371/journal.pone.0117055

**Published:** 2015-02-06

**Authors:** Cali E. Willet, Mariano Makara, George Reppas, George Tsoukalas, Richard Malik, Bianca Haase, Claire M. Wade

**Affiliations:** 1 Faculty of Veterinary Science, University of Sydney, Sydney, NSW, Australia; 2 Vetnostics, North Ryde, NSW, Australia; 3 Centre for Veterinary Education, University of Sydney, Sydney, NSW, Australia; Institut Jacques Monod, FRANCE

## Abstract

Spondylocostal dysostosis is a congenital disorder of the axial skeleton documented in human families from diverse racial backgrounds. The condition is characterised by truncal shortening, extensive hemivertebrae and rib anomalies including malalignment, fusion and reduction in number. Mutations in the Notch signalling pathway genes *DLL3*, *MESP2*, *LFNG*, *HES7* and *TBX6* have been associated with this defect. In this study, spondylocostal dysostosis in an outbred family of miniature schnauzer dogs is described. Computed tomography demonstrated that the condition mirrors the skeletal defects observed in human cases, but unlike most human cases, the affected dogs were stillborn or died shortly after birth. Through gene mapping and whole genome sequencing, we identified a single-base deletion in the coding region of *HES7*. The frameshift mutation causes loss of functional domains essential for the oscillatory transcriptional autorepression of HES7 during somitogenesis. A restriction fragment length polymorphism test was applied within the immediate family and supported a highly penetrant autosomal recessive mode of inheritance. The mutation was not observed in wider testing of 117 randomly sampled adult miniature schnauzer and six adult standard schnauzer dogs; providing a significance of association of *P*_raw_ = 4.759e^-36^ (genome-wide significant). Despite this apparently low frequency in the Australian population, the allele may be globally distributed based on its presence in two unrelated sires from geographically distant locations. While isolated hemivertebrae have been observed in a small number of other dog breeds, this is the first clinical and genetic diagnosis of spontaneously occurring spondylocostal dysostosis in a non-human mammal and offers an excellent model in which to study this devastating human disorder. The genetic test can be utilized by dog breeders to select away from the disease and avoid unnecessary neonatal losses.

## Introduction

During embryogenesis, the presomitic mesoderm generates somites, which are transient segmental structures that give rise to the axial skeleton, skeletal muscle and dermis in later development [1]. Correct formation of somite boundaries is essential to normal development. Expression of the numerous genes involved is thus tightly regulated in a spatiotemporal fashion. The role of the Notch signalling pathway in somitogenesis has been well established [2,3]. Segmentation defects of the axial skeleton in humans comprise a heterogeneous group of disorders broadly classified as spondylocostal dysostosis (SCD) or spondylothoracic dysostosis (STD). The genetic aetiology of SCD, characterized by hemivertebrae and malaligned ribs with intercostal points of fusion, has been attributed to recessively inherited mutations in four Notch genes: *DLL3* [4], *MESP2* [5], *LFNG* [6] and *HES7* [7]. Causal mutations in each of these genes give rise to further classification of SCD into types 1–4 respectively [8]. Recently, an autosomal dominant form of SCD has been attributed to *TBX6* mutation [9]. STD phenotypes display hemivertebrae and costovertebral fusions of symmetrically aligned ribs within a shortened trunk that fan out in a ‘crab-like’ arrangement. This disorder has so far only been ascribed to *MESP2* mutation [10]. While SCD is often benign, STD is lethal in an estimated 29% to 44% of patients due to respiratory insufficiency, with mortality occurring around one year of age [11,12].

The prevalence of SCD in human populations is estimated to be one in 40,000 births [13]. A survey of the peer-reviewed literature indicates that this disorder has not been described in other mammalian species, other than mutant mouse models. Spontaneously occurring SCD in a non-human animal could provide a useful model for investigation and treatment of axial skeleton dysostosis. Complex vertebral malformations in Holstein cattle have been attributed to a coding mutation in the *SLC35A3* gene [14]. This autosomal recessive disorder, also characterized by cervical and thoracic vertebral malformations including hemivertebrae, rib fusion and shortening of the trunk, provides a link between altered glycosyltransferase activity and aberrant Notch signalling [15]. In screw-tailed dog breeds, such as the pug and French bulldog, the breed-defining tail kink is caused by hemivertebrae in the tail [16]. Variably pathogenic hemivertebrae in other regions of the spine occur commonly in screw-tailed breeds [17], as well as isolated cases in other breeds including the German shorthaired pointer [18] and Dobermann pinscher [19]. While these canine disorders are thought to be inherited in an autosomal recessive fashion, no diagnosis of SCD has been made and the underlying genetic aetiology has not been identified. Our laboratory was recently presented with the opportunity to study a family of miniature schnauzer dogs in which two separate matings had produced pups with lethal skeletal malformations.

In Australia, the miniature schnauzer breed is popular due to its moderate size and agreeable temperament and in 2012 there were 1,069 pedigreed registrations [20]. The breed is predisposed to myotonia congenita, an autosomal recessive sacrolemmal channelopathy causing abnormality of gait, skull, mandible and dentition [21,22]. The musculoskeletal abnormalities displayed by the probands in this study were not consistent with myotonia congenita. While a number of other congenital genetically transmitted conditions are prevalent within the breed, such as patent ductus arteriosus and portosystemic shunt, these are not known to cause skeletal malformations. The aim of this study was to provide a clinical and genetic diagnosis for this novel disorder, which we refer to as ‘Comma defect’ due to the gross anatomical shape of the abnormal pups. We further aimed to develop a genetic test capable of establishing prevalence parameters and facilitating appropriate selection of breeding pairs to avoid producing litters bearing this disorder. Here we present the first diagnosis of SCD in an animal population, and demonstrate that the underlying mutation is a coding deletion in the *HES7* gene. A restriction fragment length polymorphism test (RFLP) of polymerase chain reaction (PCR) amplicons that can reliably distinguish carriers in the population is described. Preliminary sampling and pedigree analysis suggests that while the mutation appears to be rare amongst miniature schnauzers, the allele may be globally dispersed.

## Materials and Methods

### Samples

Of a litter of eight privately owned miniature schnauzer pups bred in Australia, three were stillborn and observed by the breeder and attending veterinarian to display abnormal body morphology. Three of the remaining five full siblings were apparently healthy and with normal phenotype, while a further two pups were stillborn but were otherwise normal phenotypically. The three morphologically abnormal stillborn pups (individual identifiers USCF134, USCF136 and USCF137) were sent to the Medical and Behavioural Genetics group, Faculty of Veterinary Science, University of Sydney for further investigation. The breeder reported that another mating using the sire of this litter produced similarly abnormal stillborn pups, but specimens from this litter were unavailable. This study was carried out in strict accordance with the recommendations in the Australian Code for the Care and Use of Animals for Scientific Purposes. Animal ethics approval for this research was granted by the University of Sydney Animal Ethics Committee (approval number N00/9–2009/3/5109, September 24 2009). All blood samples were collected by a veterinarian, and all efforts to minimize suffering during sample collection were made through the practice of appropriate animal handling skills and use of the finest gauge needle possible. The owners of the dogs gave permission for their animals to be used in this study.

### Computed tomography

To characterize the underlying skeletal morphology of abnormal stillborn pups, computed tomography (CT) scanning was carried out at the University of Sydney Veterinary Teaching Hospital using the Brilliance CT 16-Slice V3 (Philips, Amsterdam). In the absence of a normal age-matched counterpart of the same breed, an aged-matched Nova Scotia duck tolling retriever sampled for unrelated reasons was used as control (USCF32).

### Pedigree analysis

Australian National Kennel Club registered names and official pedigree transcripts were provided by the breeder, and these were used to construct a three-generation pedigree of the affected family. The pedigree was visually assessed for inheritance pattern.

### Whole genome sequencing

Two samples (USCF134; USCF136) were selected for whole genome sequencing on the Illumina HiSeq 2000 (Illumina, San Diego, CA). Tissue samples were taken from muscle of the affected pups and digested overnight with Proteinase K. Genomic DNA was extracted with the Nucleon BACC 2 Genomic DNA Extraction Kit (GE Healthcare). Paired-end libraries were prepared with the Illumina TruSeq preparation kits and sequenced according to vendor instructions by the Ramaciotti Centre at the University of New South Wales, Kensington. Libraries were barcoded and both samples sequenced in a single lane of the sequencing machine.

### Sequence alignment and variant calling

Reads were aligned as pairs to canFam2 [23] using Burrows-Wheeler Alignment (BWA) version 0.6.2 [24] with default parameters for paired-end data. Genome-wide single nucleotide polymorphisms (SNP) and insertion-deletion polymorphisms (indels) were identified using SAMtools mpileup version 1.18 [25] and filtered using custom Perl scripts. Only reads with mapping quality and bases with sequence quality of 20 or greater were included in variant detection.

### Genome-wide association mapping

Ten miniature schnauzer samples including the three affected pups, sire, dam and five additional family members were genotyped on the Canine HD BeadChip (Illumina, San Diego, CA). For the seven unaffected miniature schnauzers, genomic DNA was extracted from EDTA anti-coagulated blood samples with the Nucleon BACC 2 Genomic DNA Extraction Kit (GE Healthcare). Array genotyping was performed by GeneSeek (Lincoln, NE). Association analysis was conducted using PLINK [26], filtering out SNPs with minor allele frequency less than 0.1 and genotyping frequency less than 0.2. After frequency and genotype pruning, 73,921 SNPs remained in the analysis. Unadjusted -log10 transformed *P* values were visualised in Haploview [27]. The top 100 SNPs were examined for evidence of regional clustering. The chromosomal region harbouring the largest proportion of the top 100 SNPs was taken forward as the candidate region.

### Candidate gene and mutation identification

The candidate region was searched for potential candidate genes by first identifying the corresponding syntenic region/regions in the mouse genome. Since CT scans of the pups indicated skeletal malformation, we used the Mouse Genome Browser (http://gbrowse.informatics.jax.org/cgi-bin/gb2/gbrowse/mousebuild38/) to restrict to genes within these regions known to affect a skeletal phenotype. SNPs and indels homozygous for the non-reference allele within potential candidate genes were identified, focusing on those within exons according to the refGene and xenoRefGene tracks on the UCSC Genome Browser (http://genome.ucsc.edu/), canFam2 May 2005 build.

### PCR-RFLP genotyping

An RFLP test from PCR amplicons (PCR-RFLP) was designed to genotype samples for the candidate mutation. Primers (forward CGGAGTTGGCGATGACCA; reverse CGCTCTTCCTTTCCTGCTG) were selected using Primer3 [28] to amplify a 578 bp product flanking the mutation. PCR was carried out in a total volume of 20 μl using AmpliTag Gold 360Master Mix (Applied Biosystems) according to the manufacturer’s protocol and the product size evaluated on 1% agarose gel. PCR conditions were: heat activation for 15 minutes (mins) at 95 degrees Celsius (°C) followed by 35 cycles of 30 seconds (s) at 95°C, 30 s at 58°C and 30 s at 68°C, and terminated with a final elongation step at 72°C for 10 mins. 15 μl of PCR product was incubated with 0.3 μl of the enzyme BsrBI for 7 hours at 37°C, followed by heat inactivation. Two fragments of 472 bp and 107 bp were produced in homozygous wild type samples. The mutation removed the restriction enzyme recognition sequence (TCCGCTCC) resulting in a single uncut 578 bp fragment for homozygous affected dogs.

A total of 133 samples were genotyped using this method. 127 were miniature schnauzers, including the three affected pups and seven additional family members, with the remaining six samples from the standard schnauzer breed. For these additional dogs, blood had been collected for the routine investigation of a variety of disease conditions in two large veterinary diagnostic laboratories.

## Results

### Samples

**Figure 1:**
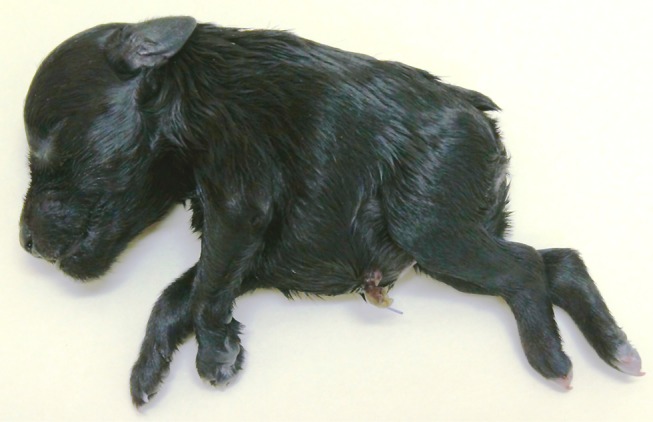
Photograph of miniature schnauzer pup USCF137 affected with Comma defect.

The three affected pups were born stillborn or died within hours of birth. Gross external examination of the pups by the attending veterinarian revealed a reduction in body length compared with normal littermates (data not available). The hindquarters of affected pups were reduced in size compared to the forequarters, giving an overall comma-like morphology to the body (Fig. 1). Individual USCF134 displayed an umbilical hernia, while USCF136 had a cleft hard palate.

### Computed tomography

**Figure 2:**
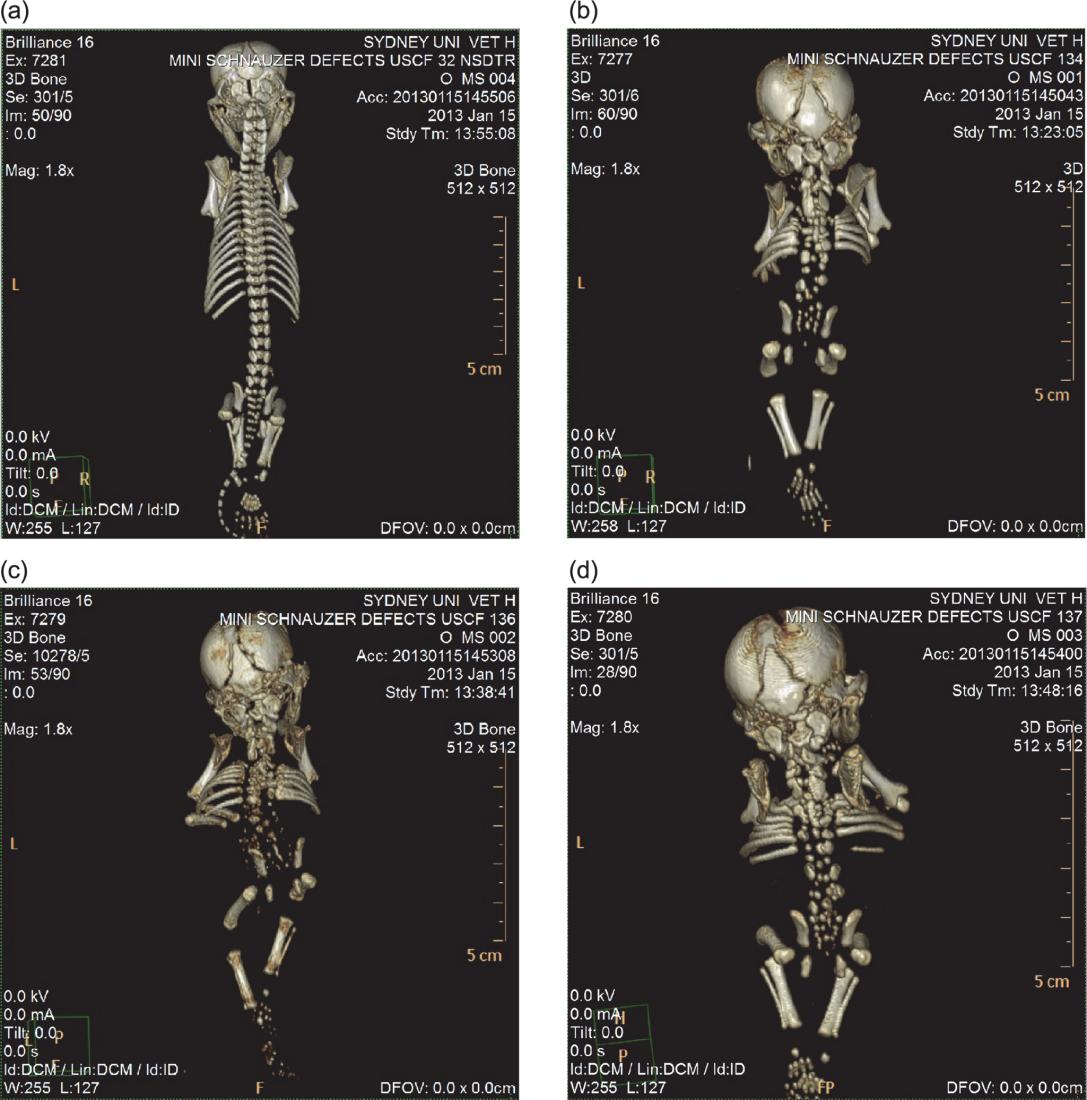
CT scans of miniature schnauzer pups affected with Comma defect and an age-matched unrelated control. (a) Unaffected Nova Scotia duck tolling retriever pup sampled for unrelated reasons. (b) Affected miniature schnauzer pup USCF134. (c) Affected miniature schnauzer pup USCF136. (d) Affected miniature schnauzer pup USCF137.

All three affected miniature schnauzer pups displayed severe morphological defects of the axial skeleton (Fig. 2). Sacrococcygeal agenesis was observed in addition to fully segmented hemivertebrae in the cervical, thoracic and lumbar regions. The rib cage was poorly developed with a reduced number of ribs displaying malalignment and sporadic lateral fusions. Since the probands were frozen after death, soft tissue imaging was unable to clearly depict the physical state of organs and cartilage.

### Pedigree analysis

**Figure 3:**
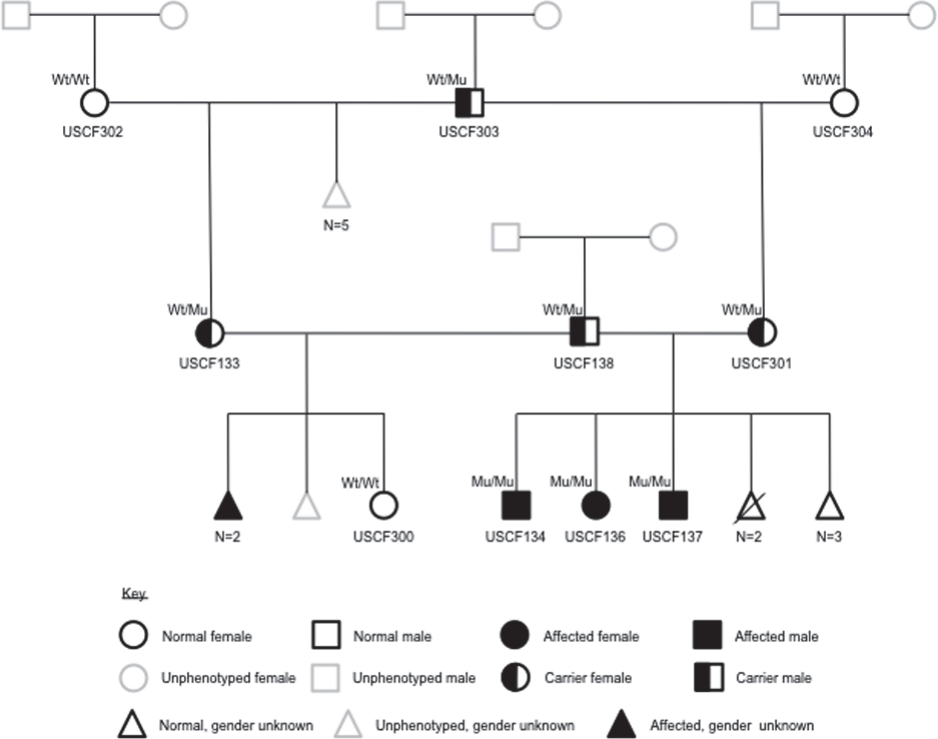
Three-generation pedigree for the miniature schnauzer family producing litters affected with Comma defect. Individuals with USCF identifiers (*n* = 10) are those that have been phenotyped and for which DNA samples are available. Genotypes as determined by PCR-RFLP are indicated (Wt = wild type; Mu = mutant). Unsampled animals are depicted by grey outlines.

The pattern of inheritance within the three-generation pedigree was consistent with a highly penetrant autosomal recessive monogenic trait (Fig. 3). The reported relationships were supported by identity by descent (IBD) estimation using PLINK (S1 Table).

### Genome-wide association mapping

**Figure 4:**
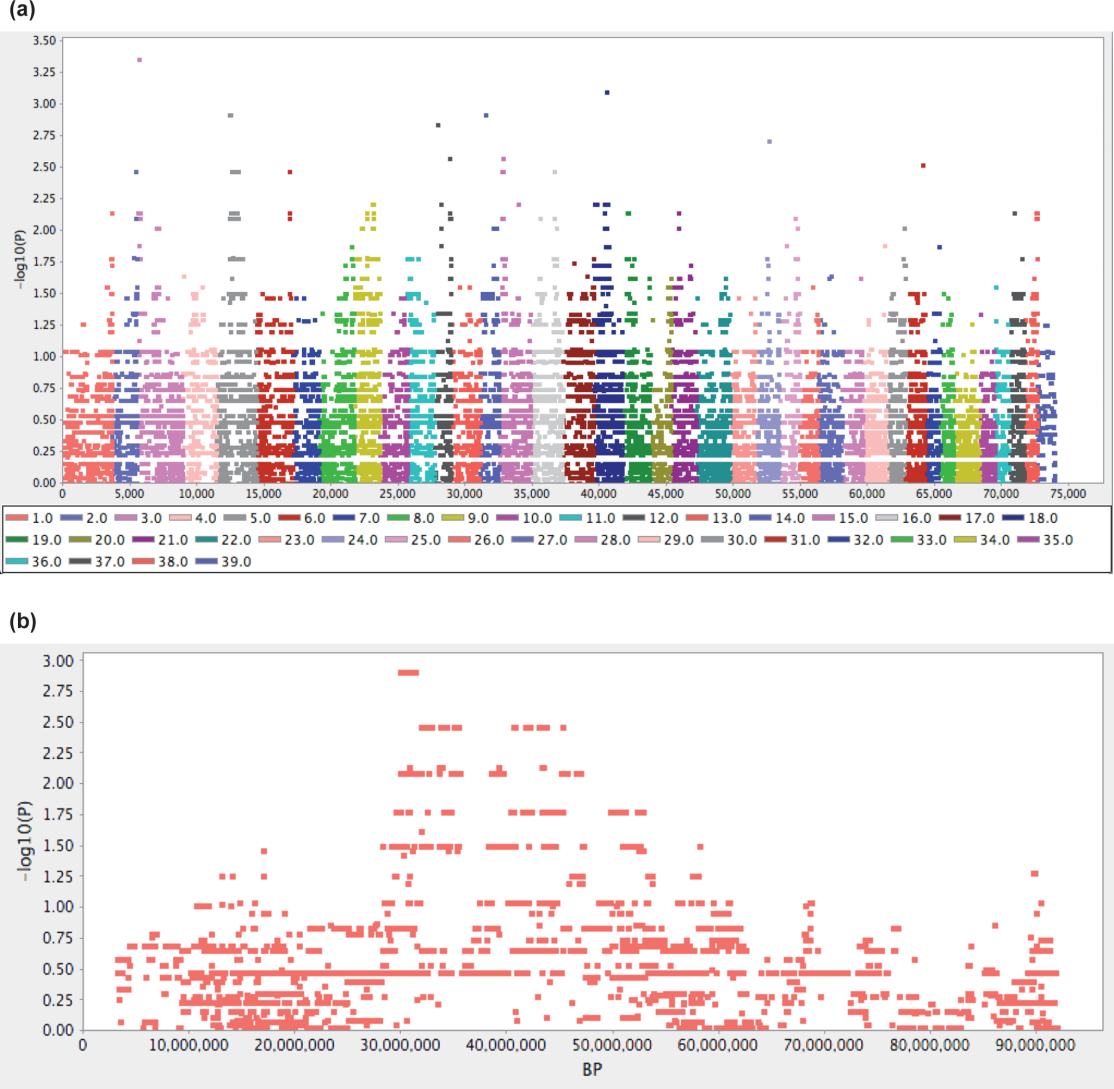
Genome-wide association analysis for Comma defect. The analysis included three cases and seven controls genotyped on the Canine HD BeadChip. Coordinates shown are from the canFam2 assembly. (a) Haploview plot of -log_10_ transformed *P* values for 73,921 SNPs tested for association using PLINK. (b) Haploview plot of -log_10_ transformed *P* values for SNPs on CFA5.

PLINK genome-wide association analysis using ten individuals was hindered by limited statistical power, and this was evident when the results visualised with Haploview showed no strong signal of association (Fig. 4A). We therefore approached the PLINK data using an alternate method, selecting the 100 most significant SNPs and identifying which chromosomes were overrepresented in this group. Of the 100 most significant SNPs, 60% resided on chromosome 5 (CFA5), 13% on CFA18 and 8% on CFA12. Haploview plotting of all SNPs within chromosome demonstrated a dispersed but contiguous signal of around 25 megabases (Mb) on CFA5 (Fig. 4B). Markers with unadjusted *P* values less than 0.005 refined the peak association to CFA5:29.84 Mb–45.26 Mb.

### Candidate gene and mutation analysis

Genes that have been linked to phenotypic groups in mouse models are a useful way of reducing large gene lists to a handful of candidates. The associated region was found to be syntenic with two mouse chromosomes on build GRCM38. CFA5:29.84 Mb–33.1 Mb was broadly syntenic with mouse chromosome 9:5.29 Mb–8.54 Mb, and CFA5:33.1 Mb–45.26 Mb with mouse chromosome 11:59.6 Mb–72.8 Mb. Searching these two regions for genes with a known abnormal skeletal phenotype in the Mouse Genome Browser restricted the number of candidate genes to 19. From a total of 27,724 SNPs and 5,808 indels identified from the sequence alignment of the affected samples within the candidate region, 274 SNPs and 12 indels resided within exons. Filtering these putatively coding variants to those occurring within one of the 19 genes with reported involvement in skeletal malformations in mice, four SNPs and one indel were observed (Table 1). Of these genes, mutations in *Hes7* and *Tmem107* have been linked to phenotypes in mice with similarities to the affected pups in this study. A non-conservative substitution within *TMEM107* was excluded as a candidate due to presence in the homozygous state in whole genome sequence datasets of other breeds sequenced by our laboratory. A guanine deletion at CFA5:35,940,090 (CFA5:32,945,846 in canFam3.1) within exon 2 of *HES7* (c.126delG) was taken forward for further analysis.

**Table 1:** List of candidate functional mutations observed in genome sequence of pups affected with Comma defect.

canFam2 CFA5 location (bp)	Polymorphism	Gene	Skeletal phenotype	USCF134 genotype	USCF136 genotype	Impact on protein
35,940,090	Deletion, C	HES7	Abnormal rib and vertebrae morphology, decreased rib number, rib fusion, postnatal lethality (J:155378; J:72325; J:184024)	Del/Del (0/3)	Unknown (0/0)	Frameshift
35,954,496	SNP, G→T	PER1	Increased osteoblast cell number, increased bone mass (J:115188)	TT (0/4)	TT (0/2)	Serine to arginine
35,985,813	SNP, C→A	TMEM107	Abnormalities to bones of the skull, sternum and ribs (J:186552)	AA (0/14)	AA (0/5)	Arginine to leucine
36,235,602	SNP, C→T	MYH10	Abnormal cranium morphology, micrognathia (J:175213); domed cranium (J:44181)	TT (0/3)	Unknown (0/1)	Valine to isoleucine
37,777,975	SNP, T→C	MYH1	Kyphosis (J:44448)	CC (0/2)	CC (0/5)	Isoleucine to valine

JBrowse references are provided for each mutant mouse skeletal phenotype. The number of quality sequence reads supporting the reference and alternate allele respectively are shown in brackets for each genotype.

The predicted canine *HES7* mRNA sequence was obtained from NCBI (XM_844962.3). An unknown nucleotide (N) present within the predicted mRNA sequence along with the three preceding bases was corrected using the sequence data of USCF134 (S1 Dataset). The wild-type canine HES7 protein is predicted to be 224 amino acids in length. The mutation was introduced into this sequence and translated with the ExPASy translate tool (http://web.expasy.org/translate/) (S1 Dataset). The deletion introduces a frameshift mutation, causing alteration from the 43^rd^ amino acid onwards and resulting in a premature termination codon in place of the 66^th^ amino acid (p.(Thr43ProfsTer24)). The Conserved Domain Database (http://www.ncbi.nlm.nih.gov/Structure/cdd/cdd.shtml) was used to identify important features in the predicted wild-type protein. Significant hits were detected at residues 14–68 (Helix-loop-helix DNA-binding domain; pfam00010) and at residues 91–128 (Hairy Orange domain; pfam07527). Both of these functional sites are affected by the candidate deletion; the terminal half of the helix-loop-helix (HLH) domain being altered and the Hairy Orange domain being completely absent from the predicted mutant protein sequence.

### PCR-RFLP genotyping

**Figure 5:**
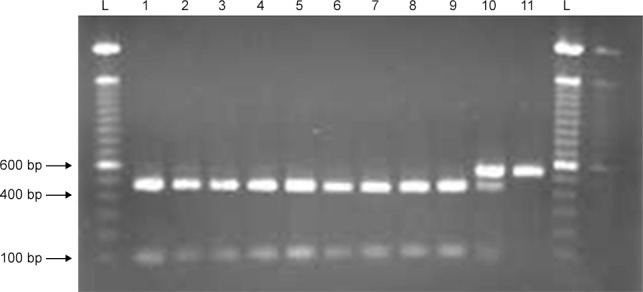
Comma defect PCR-RFLP test results for 11 miniature schnauzers. Samples in lanes 1–9 are homozygous for the wild-type genotype; lane 10 shows a carrier for the deletion; lane 11 is homozygous for the mutant allele. L = ladder (100 bp).

The PCR-RFLP test was designed to detect carriers by the presence of three bands, as opposed to two bands (472 bp and 107 bp) for homozygous wild type and a single uncut 578 bp fragment for homozygous deleted (Fig. 5). Of 133 samples tested, only the three proband pups tested homozygous for the deletion. Both parents of this litter tested heterozygous, as did the maternal grandsire and another of his female offspring. These within-family genotyping results support an autosomal recessive mode of inheritance (Fig. 3). All remaining 126 samples tested homozygous for the wild type allele. The Chi-squared value for association at this locus, X^2^ (2, N = 133) = 157, gives a genome-wide significant raw probability of 4.759e^-36^ [25].

## Discussion

Careful research into the expression of basic HLH genes and their influence on somitogenesis by Bessho *et al*. [29,30] discovered Hes7 in the presomitic mesoderm of mouse embryos. Expression was found to be specific to the presomitic mesoderm, controlled by Notch signalling and oscillating in a two-hour cycle. The cyclic expression of HES7 is achieved through autoregulation: the basic domain of HES7 protein binds DNA via an N-box, while the HLH region can heterodimerize with E47 transcription factor preventing interaction with E-boxes [7,29,31]. Knockout experiments demonstrated that *H*es*7*-deficient mice had severe segmentation defects of the axial skeleton, confirming the essential role of Hes7 in somitogenesis [30]. Later work established the critical importance of Hes7 autoregulation in the segmentation clock [32].

Since then, *HES7* mutations have been identified in a handful of human SCD cases, giving rise to the disease subtype SCD4. A SNP in exon 2 (c.73C>T) created a substitution (p.Arg25Trp) at a conserved site essential for DNA binding [7]. Functional analysis *in vitro* demonstrated impaired transcriptional repression activity through both diminished affinity of the mutant HES7 protein to bind promoter N-box sequence and to heterodimerize with E47. Compound heterozygosity for two non-synonymous SNPs in exon 3 (c.172A>G, p.Ile58Val) and exon 4 (c.556G>T, p.Asp186Tyr) was identified in two siblings with SCD4 from a non-consanguineous family [33]. While only the substitution in exon 4 impaired transcriptional repression ability of the mutated HES7 protein, both mutations were required to cause segmentation defects *in vivo*. In contrast to these cases where the patients survived, a 10-base duplication in exon 4 (c.400_409dupAAACCGCCCC, p.Arg137GlnfsTer42) resulted in infant mortality in two of seven affected individuals [34].

The frameshift mutation reported in the present study affects the same exon as that described in [7], yet the severity of the phenotype differs. The human patient presented with a number of syndromes but only minor motor and growth retardation. In contrast, the pups in this study were born stillborn or died within hours of birth. Similar to the deceased cases in [34], mortality was likely due to impaired respiratory function resulting from truncal shortening and rib fusion. This differential prognosis is hardly surprising, given the more extreme changes at the protein level induced by frameshift mutations compared to SNPs. Where the SNP affected a single residue of the HLH domain, the indel affected multiple residues and eventually caused premature termination of the protein. The transcriptional repression ability of the mutant protein in our study would thus be attenuated or completely lost, preventing essential downregulation of HES7 required for somite boundary formation. While the indel described in [34] occurs later in the mRNA sequence, the increased disruptiveness of a frameshift mutation is reflected by the mortality rate of 29%. In both the present study and [34], the position of the premature termination codon within the mRNA transcript would not enable nonsense-mediated decay to prevent translation of the mutant mRNA into protein [35], supporting that the deleterious effects of these mutations result from loss-of-function rather than gain-of-function. While previous work did not find HES7 stability or expression changes due to additional missense amino acids [34], the impact of the 23 missense amino acids in the mutated HES7 in the present study could alter protein stability or expression levels. Such changes if present would be unlikely to alter the outcome for the affected pups in this case, but may potentially influence phenotype in heterozygous individuals.

Haploinsufficiency has been reported in the literature for *HES7* mutations. Kinked tails were observed in 43% of heterozygous *Hes7*-knockout mice [30], and later investigation of the developing axial skeleton of *Hes7*+/- mouse embryos found 53% with vertebral defects ranging from mild to severe [36]. In this study, a human patient with scoliosis was found to be heterozygous for a non-synonymous SNP in *HES7* that impaired the autorepressive activity of the protein. The proband’s mother, also heterozygous for the SNP, did not display external signs of vertebral malformation, nor did carrier family members of SCD4-affected individuals in other studies, results which were further confirmed by radiograph [7,34]. These data suggest that the penetrance of *HES7* haploinsufficiency is incomplete. In the present study, three carriers were identified. These are registered show dogs with no external signs of vertebral malformation, although one carrier has a docked tail which would prevent the detection of tail kinking if present. Due to ethical constraints, we were unable to obtain radiographs of these dogs for vertebral analysis. The potential for aberrant skeletal phenotypes to occur in dogs heterozygous for *HES7* and other Notch-related gene mutations should be considered in the veterinary clinical setting as well as in further canine SCD genetic investigations.

It is evident from human SCD studies that while penetrance of the vertebral defects is complete, the severity of the condition and presence of concomitant syndromes varies even between patients with the same mutation. For example, three of seven patients with the same *HES7* mutation displayed dextrocardia with *situs inversus* [34], while this syndrome was absent among other SCD4 patients [7,33]. Although it could not be determined whether the probands in this study presented with dextrocardia or any of the other syndromes described in [7,33,34] due to the frozen state of the pups hindering soft tissue image clarity, umbilical hernia and cleft palate were detected. Umbilical hernia is observed in both SCD and STD, but cleft palate is associated with STD but not SCD [37]. Study of the physiological difference between SCD4 phenotype in dogs compared to humans may shed further light on the complex interaction of multiple genes required for normal somitogenesis and embryonic development. Dogs are an ideal model species for human disease [38,39]. Hundreds of spontaneously occurring congenital disorders analogous to human diseases have been identified. Disease heterogeneity is limited due to within-breed homogeneity following centuries of selective breeding for specific traits. Further, dogs generally share the same home environment and levels of physical activity as their owners and receive roughly comparable levels of medical care. Whilst the importance of mouse models to gene and disease research is indisputable, the biological and epidemiological similarities between human and canine disease cases makes dog a superior model system in which to study aspects such as genomic instability, gene-environment interaction and long term treatment outcomes. As dogs are larger than mice, certain procedures such as CT and ultrasonography are possible *in utero*. Further, since these disorders are occurring in a natural population, there is no need to create and maintain a study colony, and the efforts which are invested to study the disorder for the benefit of human health simultaneously benefits canine health and welfare through increased understanding, diagnosis and treatment of the disease.

The predisposition towards inherited diseases in pedigree dogs is an area of strong public opinion. The PCR-RFLP test developed in this study can be used to accurately detect carriers for SCD4 in potential breeding animals, enabling breeders to reduce the incidence of neonatal mortality. The modest number of miniature schnauzer and standard schnauzer dogs sampled in this study suggests that SCD4 is rare, with carriers only detected within the immediate family. However, the pedigree is outbred, with imported animals from America and Europe contributing the recessive alleles on the sire and dam sides of the pedigree, respectively. The dams of the two affected litters (USCF301; USCF133) are half-siblings through a Swedish imported sire (USCF303), while the common sire of the affected litters (USCF138) was imported from Argentina. The respective imported sires were unrelated for more than four generations. This information suggests that the mutant recessive allele is globally dispersed. Among multiparous mammals, some percentage of neonatal loss or abnormality is considered normal. It is likely that this litter is not the first to be affected with SCD4, and that breeders were unable to appreciate the nature of the problem since the disorder was previously uncharacterized in dogs. The fact that we were able to successfully map the region using markers in the canine array, which were selected to be polymorphic in a number of dog breeds, supports that this mutation is not evolutionarily new and may have been present in the miniature schnauzer breed, and potentially other breeds, for many generations.

By publicizing the first case of SCD4 in dogs, we equip breeders and their veterinarians with the knowledge required to identify potential cases and verify the diagnosis by PCR-RFLP test. Wider testing of miniature schnauzers on an international scale is recommended to obtain more comprehensive prevalence parameters and increase breeder awareness of canine SCD4. It would also be useful to apply this test to breeds in which pathogenic hemivertebrae are observed. We refer to the disorder as Comma defect, terminology chosen to aid breeders in associating this characteristic abnormal body shape in stillborn pups with a disorder for which a reliable genetic test is available.

## Supporting Information

S1 Dataset Predicted wild-type and mutant canine *HES7* mRNA and protein sequences. (DOCX)

S1 Table Relationship estimation from genotypes. (DOCX)
