# Supplementary material for: Canine Disorder Mirrors Human Disease: Exonic Deletion in HES7 Causes Autosomal Recessive Spondylocostal Dysostosis in Miniature Schnauzer Dogs
Source: PLoS One. 2015 Feb 6;10(2):e0117055. doi: 10.1371/journal.pone.0117055 (PMC4319916; doi:10.1371/journal.pone.0117055)
Supplement: S1 Dataset — (DOCX) [file pone.0117055.s001.docx]

**Dataset S1. Predicted wild-type and mutant canine *HES7* mRNA and protein sequences.** The predicted canine *HES7* mRNA was obtained from NCBI (XM_844962.3). This sequence contains an unknown nucleotide (N) at position 163 from coding start; there is no base present at this location within the canFam2 reference sequence. Sequence reads obtained from sample USCF134 covering this location were used to identify the correct sequence at this position and supported a sequence of ‘AAAG’ as opposed to ‘NTTT’ at this location (relative to the reverse strand of the canFam2 reference sequence, consistent with the direction of gene coding). We inserted the deletion (c.126delG) into the amended predicted wild-type sequence. Both sequences and their predicted translations are shown below.

> wild-type_mRNA

ATGGTCACTCCGGATCCAGCGGAGAATAGGGACGGCCCCAAGATGCTGAAGCCGCTGGTGGAGAAGCGGCGCCGGGACCGCATCAACCGCAGCCTGGAAGAGCTGAGGCTGCTGCTGCTGGAGCGGACCCGGGACCAGAACCTCCGCAACCCGAAGCTGGAGAAAGCAGAGATACTGGAGTTCGCCGTGGGCTACTTGAGGGAGCCGAGCCGGGTGGAGCCCCCGGGGGTTCCCCGGTCCCCAGCTCAGGACGCCGAGGCGCTCGCCAGCTGCTACTTGTCCGGCTTCCGCGAGTGCCTGCTTCGCCTGGCGGCCTTCGCGCACGACGCCAGCCCGGCCGCCCGCGCCCAGCTCTTCTCCGCGCTGCACGGCTACCTGCGCCCCAAGCCGCCCCGGCCGGAGCCGGTAGATCCGAGGCCCCAAGCGCCTCGCCCTCCGCTGGACCCCGCCGCCCCGGCGCCCGGCCCCGCGCTGCACCAGCGCCCCCCAGTGCACCAGGGCCCCCGTAGCCCGCGCTGCGCCTGGTCCCCGTCCCCCTGCTCGCCCCGCGCCGGGGATCCCGGCGTGCCGGCGCCCCTCACCGGACTGCTGCCGCCGCCGCCGCCGCACAGACAAGACGGGGCGCCCAAGGCCCCGCCGCCCCCGCCACCCGCTTTCTGGAGACCTTGGCCCTGA

> mutant_mRNA

ATGGTCACTCCGGATCCAGCGGAGAATAGGGACGGCCCCAAGATGCTGAAGCCGCTGGTGGAGAAGCGGCGCCGGGACCGCATCAACCGCAGCCTGGAAGAGCTGAGGCTGCTGCTGCTGGAGCGACCCGGGACCAGAACCTCCGCAACCCGAAGCTGGAGAAAGCAGAGATACTGGAGTTCGCCGTGGGCTACTTGAGGGAGCCGAGCCGGGTGGAGCCCCCGGGGGTTCCCCGGTCCCCAGCTCAGGACGCCGAGGCGCTCGCCAGCTGCTACTTGTCCGGCTTCCGCGAGTGCCTGCTTCGCCTGGCGGCCTTCGCGCACGACGCCAGCCCGGCCGCCCGCGCCCAGCTCTTCTCCGCGCTGCACGGCTACCTGCGCCCCAAGCCGCCCCGGCCGGAGCCGGTAGATCCGAGGCCCCAAGCGCCTCGCCCTCCGCTGGACCCCGCCGCCCCGGCGCCCGGCCCCGCGCTGCACCAGCGCCCCCCAGTGCACCAGGGCCCCCGTAGCCCGCGCTGCGCCTGGTCCCCGTCCCCCTGCTCGCCCCGCGCCGGGGATCCCGGCGTGCCGGCGCCCCTCACCGGACTGCTGCCGCCGCCGCCGCCGCACAGACAAGACGGGGCGCCCAAGGCCCCGCCGCCCCCGCCACCCGCTTTCTGGAGACCTTGGCCCTGA

>wild-type_protein

MVTPDPAENRDGPKMLKPLVEKRRRDRINRSLEELRLLLLERTRDQNLRNPKLEKAEILEFAVGYLREPSRVEPPGVPRSPAQDAEALASCYLSGFRECLLRLAAFAHDASPAARAQLFSALHGYLRPKPPRPEPVDPRPQAPRPPLDPAAPAPGPALHQRPPVHQGPRSPRCAWSPSPCSPRAGDPGVPAPLTGLLPPPPPHRQDGAPKAPPPPPPAFWRPWP

>mutant_protein

MVTPDPAENRDGPKMLKPLVEKRRRDRINRSLEELRLLLLERPGTRTSATRSWRKQRYWSSPWAT
