# Supplementary material for: Canine Disorder Mirrors Human Disease: Exonic Deletion in HES7 Causes Autosomal Recessive Spondylocostal Dysostosis in Miniature Schnauzer Dogs
Source: PLoS One. 2015 Feb 6;10(2):e0117055. doi: 10.1371/journal.pone.0117055 (PMC4319916; doi:10.1371/journal.pone.0117055)
Supplement: S1 Table — (DOCX) [file pone.0117055.s002.docx]

**Table S1. Relationship estimation from genotypes.**

| ID1 | ID2 | Relationship | Expected IBD | Proportion IBD |
| --- | --- | --- | --- | --- |
| USCF133 | USCF134 | OT | 0.125 | 0.0877 |
| USCF133 | USCF136 | OT | 0.125 | 0.1099 |
| USCF133 | USCF137 | OT | 0.125 | 0.1056 |
| USCF133 | USCF138 | OT | 0 | 0.0658 |
| USCF133 | USCF300 | PO | 0.5 | 0.4996 |
| USCF133 | USCF301 | HS | 0.25 | 0.1738 |
| USCF133 | USCF302 | PO | 0.5 | 0.5 |
| USCF133 | USCF303 | PO | 0.5 | 0.5 |
| USCF133 | USCF304 | OT | 0 | 0 |
| USCF134 | USCF136 | FS | 0.5 | 0.534 |
| USCF134 | USCF137 | FS | 0.5 | 0.4497 |
| USCF134 | USCF138 | PO | 0.5 | 0.5039 |
| USCF134 | USCF300 | HS | 0.25 | 0.2503 |
| USCF134 | USCF301 | PO | 0.5 | 0.5612 |
| USCF134 | USCF302 | OT | 0 | 0 |
| USCF134 | USCF303 | OT | 0.25 | 0.3223 |
| USCF134 | USCF304 | OT | 0.25 | 0.2775 |
| USCF136 | USCF137 | FS | 0.5 | 0.5721 |
| USCF136 | USCF138 | PO | 0.5 | 0.512 |
| USCF136 | USCF300 | HS | 0.25 | 0.1951 |
| USCF136 | USCF301 | PO | 0.5 | 0.5371 |
| USCF136 | USCF302 | OT | 0 | 0 |
| USCF136 | USCF303 | OT | 0.25 | 0.3674 |
| USCF136 | USCF304 | OT | 0.25 | 0.1472 |
| USCF137 | USCF138 | PO | 0.5 | 0.5365 |
| USCF137 | USCF300 | HS | 0.25 | 0.2886 |
| USCF137 | USCF301 | PO | 0.5 | 0.5032 |
| USCF137 | USCF302 | OT | 0 | 0 |
| USCF137 | USCF303 | OT | 0.25 | 0.3246 |
| USCF137 | USCF304 | OT | 0.25 | 0.1602 |
| USCF138 | USCF300 | PO | 0.5 | 0.5077 |
| USCF138 | USCF301 | OT | 0 | 0.0698 |
| USCF138 | USCF302 | OT | 0 | 0 |
| USCF138 | USCF303 | OT | 0 | 0.1508 |
| USCF138 | USCF304 | OT | 0 | 0 |
| USCF300 | USCF301 | OT | 0.125 | 0.0721 |
| USCF300 | USCF302 | OT | 0.25 | 0.2476 |
| USCF300 | USCF303 | OT | 0.25 | 0.259 |
| USCF300 | USCF304 | OT | 0 | 0 |
| USCF301 | USCF302 | OT | 0 | 0 |
| USCF301 | USCF303 | PO | 0.5 | 0.5177 |
| USCF301 | USCF304 | PO | 0.5 | 0.5 |
| USCF302 | USCF303 | OT | 0 | 0 |
| USCF302 | USCF304 | OT | 0 | 0 |
| USCF303 | USCF304 | OT | 0 | 0 |

Note: Samples were genotyped on the Canine HD BeadChip. Relationships were estimated using the IBD estimation function in PLINK. Genotypes were filtered for minor allele frequency greater than 0.1 and genotyping rate greater than 0.2. PO = parent/offspring; FS = full siblings; HS = half siblings; OT = other related.
